# Supplementary material for: The splanchnic mesenchyme is the tissue of origin for pancreatic fibroblasts during homeostasis and tumorigenesis
Source: Nat Commun. 2023 Jan 3;14:1. doi: 10.1038/s41467-022-34464-6 (PMC9810714; doi:10.1038/s41467-022-34464-6)
Supplement: Supplementary file 1 — Supplementary Information [file 41467_2022_34464_MOESM1_ESM.pdf]

The splanchnic mesenchyme is the tissue of origin for pancreatic fibroblasts during homeostasis and tumorigenesis

Lu Han[1], Yongxia Wu[2,5], Kun Fang[3], Sean Sweeney[1], Ulyss K. Roesner[1], Melodie Parrish[1], Khushbu Patel[1], Tom Walter[1], Julia Piermattei[1], Anthony Trimboli[4,5], Julia Lefler[1], Cynthia D. Timmers[1], Xue-Zhong Yu[2,5], Victor X. Jin[3], Michael T. Zimmermann[4,5,6,7], Angela J. Mathison[6,8], Raul Urrutia[4,5,6,8], Michael C. Ostrowski[1,*], Gustavo Leone[4,5,*]

[1]Department of Biochemistry and Molecular Biology, Hollings Cancer Center, Medical University of South Carolina, 171 Ashley Ave, Charleston, SC 29425, USA.

[2]Department of Microbiology and Immunology, Hollings Cancer Center, Medical University of South Carolina, 171 Ashley Ave, Charleston, SC 29425, USA.

[3]Division of Biostatistics and Cancer Center, Medical College of Wisconsin, 8701 Watertown Plank Road, Milwaukee, WI 53226, USA.

[4]Department of Biochemistry, Medical College of Wisconsin, 8701 Watertown Plank Road, Milwaukee, WI 53226, USA.

[5]Medical College of Wisconsin Cancer Center, Medical College of Wisconsin, 8701 Watertown Plank Road, Milwaukee, WI 53226, USA.

[6]Linda T. and John A. Mellowes Center for Genomic Sciences and Precision Medicine, Medical College of Wisconsin, Milwaukee, WI 53226, USA.

[7]Clinical and Translational Sciences Institute, Medical College of Wisconsin, Milwaukee, WI 53226, USA.

[8]Department of Surgery, Medical College of Wisconsin, 8701 Watertown Plank Road, Milwaukee, WI 53226, USA.

* Co-corresponding: gleone@mcw.edu; ostrowsk@musc.edu

Supplementary Figures 1-8

Supplementary Figure 1

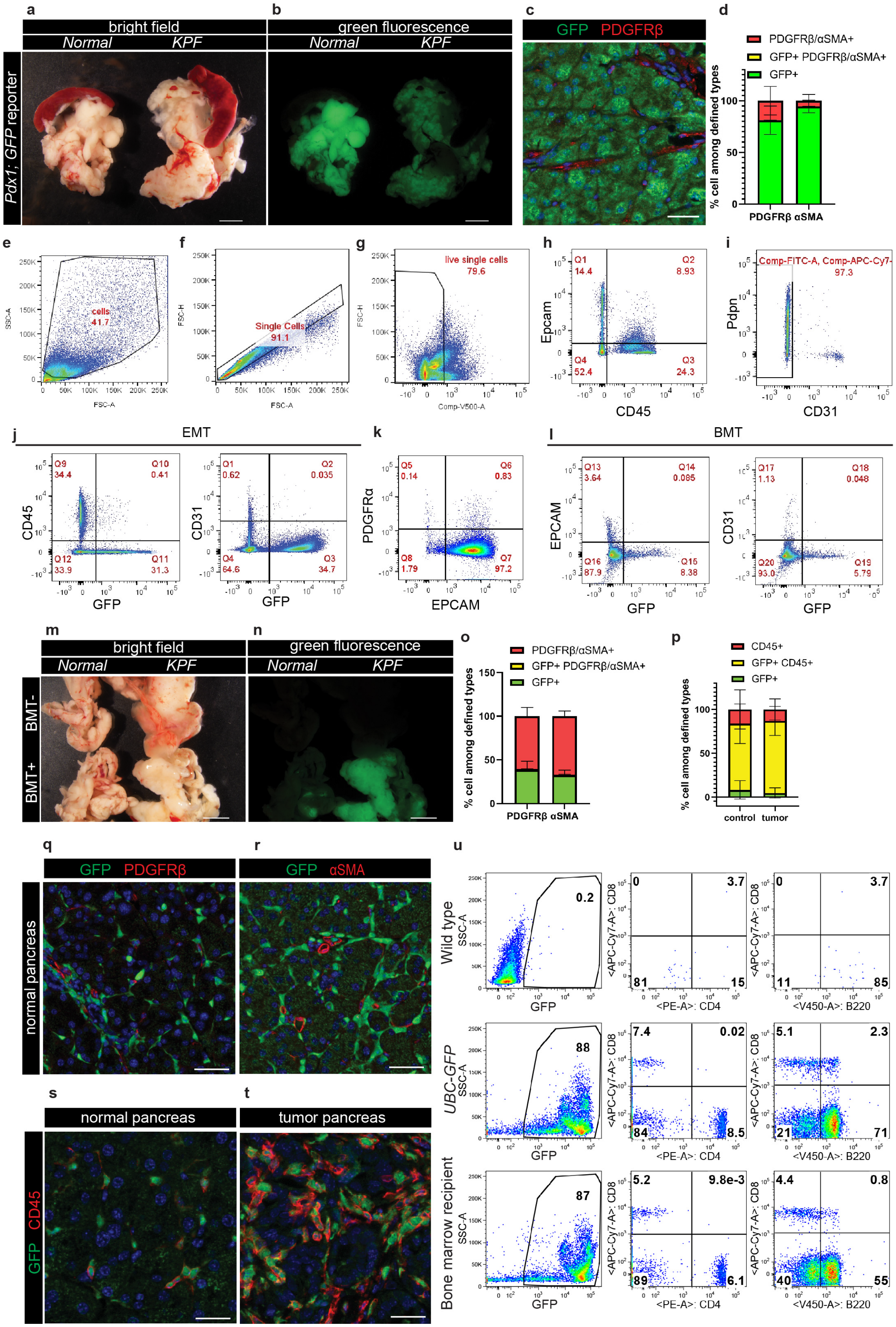

Supplementary Fig. 1. Epithelium and bone marrow cells rarely become fibroblasts in both normal and tumor bearing pancreas. a-b, Bright field and green epifluorescence views of dissected pancreata and spleens. normal n=3 mice; tumor n=14 mice. **c-d**, Co-immunostaining on the pancreas and quantification of cells that are either single positive or double positive for defined markers. n=6 mice for PDGFRβ; n=4 mice for αSMA. **e-i**, Flow cytometry gating strategy to identify the fibroblast population by negatively selecting other cell markers**. j**, Flow cytometry analysis of dissociated pancreata from a KPFG mouse. **k**, Flow cytometry analysis of gated GFP+ cells from dissociated KPFG pancreata. **l**, Flow cytometry analysis of dissociated pancreata from a KPF recipient of GFP+ bone marrow. m**-n**, Bright field and green epifluorescence views of dissected pancreata and spleens. No transplant group, n=8 mice; normal + transplant group, n=14 mice; tumor + transplant group, n=15 mice. **o-p**, Quantification of cells that are either single positive or double positive for defined markers in images represented in **q-t**. In **o**, n=5 mice for PDGFRβ; n=3 mice for αSMA. In **p**, n=4 mice for "normal"; n=5 mice for "tumor". **q-t**, Co-immunostaining of the pancreatic tissue from normal mice transplanted with GFP+ bone marrow. **u**, Flow cytometry analysis of the peripheral blood. Plots in the second and third columns were gated GFP+ cells from the plots in the first column. Bone marrow transplant recipients were harvested five months after transplantation. Data are mean ± SD. Scale bars in **a, b, m** and **n**: 1000μm; in **c** and **q-t**: 30 μm. Source data are provided as a Source Data file.

Supplementary Figure 2

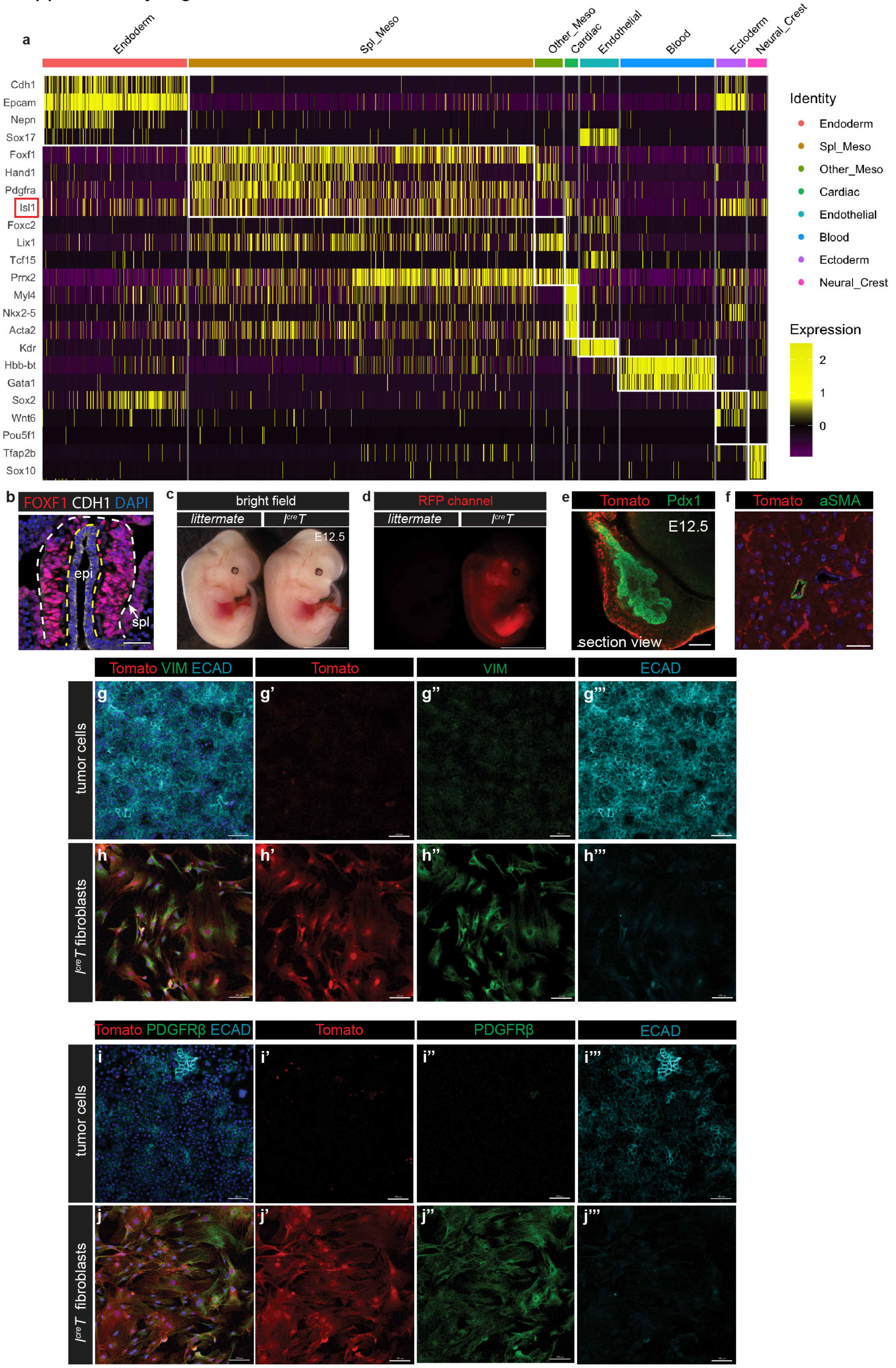

Supplementary Fig. 2. ***I^{cre}T* targeted mesenchyme during fetal development and fibroblasts derived from the pancreata. a,** Heatmap of expressions of known markers for different cell lineages. Each column represents an individual cell within different clusters. **b**, Immunostaining on transverse sections across the foregut level of E9.5 mouse embryos. Yellow dashed lines delineate the foregut epithelium and the region between the yellow and the white dashed lines delineate the splanchnic mesenchyme. n=3 embryos. **c-d**, Bright field and green epifluorescence views of dissected embryos of $I^{cre}T$ genotype and their littermate. Littermate n = 4 embryos; $I^{cre}T$ n = 5 embryos. **e**, Section view of whole mount stained fetal pancreas. **f**, Co-immunostaining of Tomato and αSMA on pancreatic tissues from $I^{cre}T$ mice. **g-j**, Immunostaining on cultured fibroblasts from $I^{cre}T$ adult pancreas and tumor cells derived from the *KPF* mice. Scale bars for b: 50 μm; for c-d: 1000 μm, for e-j: 100μm. Spl, splanchnic; meso, mesoderm; epi, epithelium.

Supplementary Figure 3

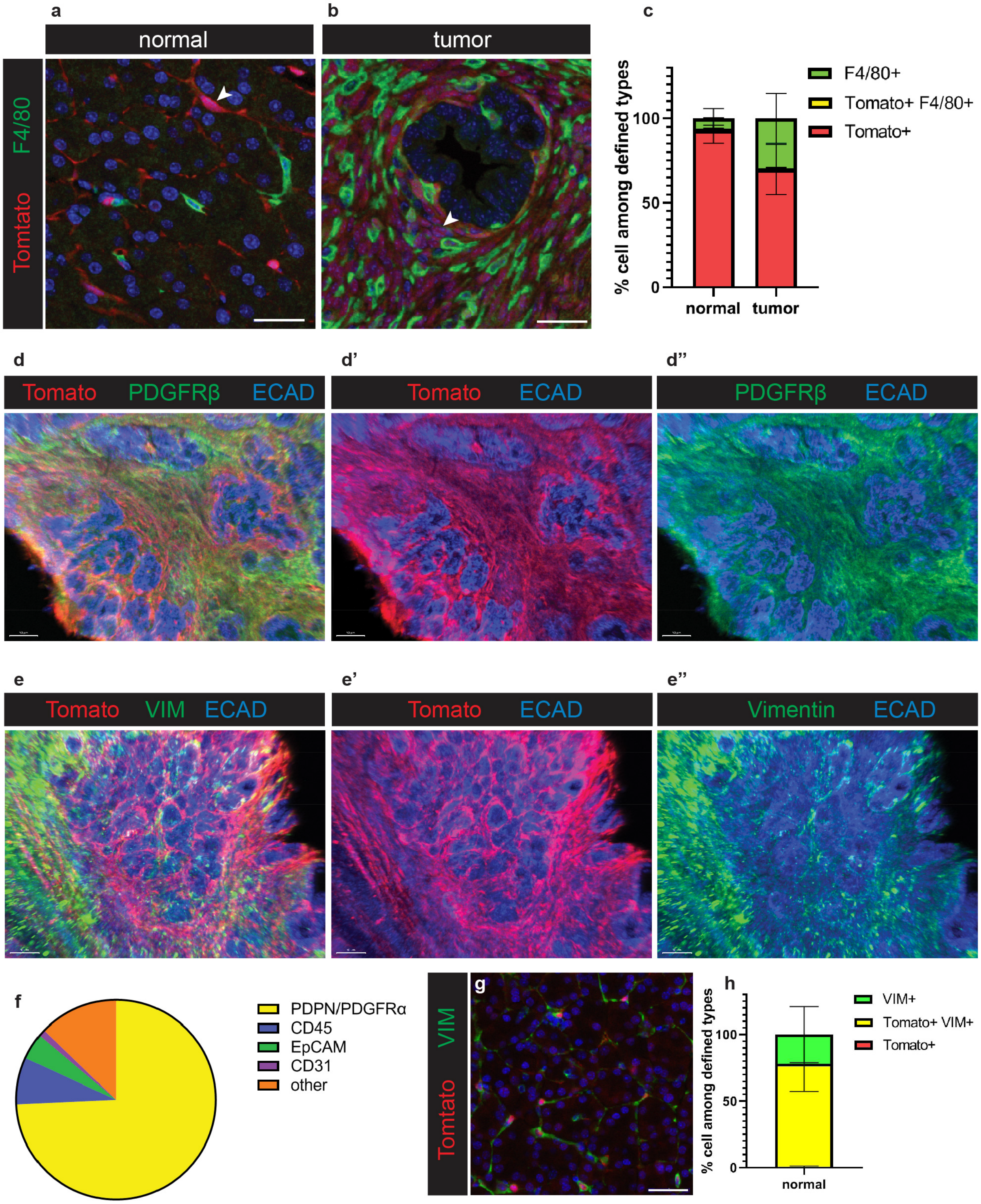

Supplementary Fig. 3. *KPFI^{cre}T* targeted cells contribute to fibroblasts, but not macrophages in the adult pancreas. a-b, Co-immunostaining of adult pancreas from either *I^{cre}T* or *KPFI^{cre}T* mice. **c,** Quantification of cells that are either single positive for Tomato or F4/80, or double positive for Tomato and F4/80 in images represented in **a** and **b**. n = 5 mice for *I^{cre}T* (normal); n = 5 mice for the *KPFI^{cre}T* mice. Data are mean ± SD. **d-e,** whole mount immunostaining of thin slices of *KPFI^{cre}T* tumor pancreas. n=1 mouse for each stain panel. **f**, Flow cytometry quantification of the percentage of Tomato positive cells that express each cell type markers. n = 4 mice. **g**, Immunostaining of an apparently normal region in a *KPFI^{cre}T* pancreas harvested at an early stage (30 day). **h**. Quantification of cells in images represented in g. n=3 mice. Data are mean ± SD. Scale bars in **a-b**, g, 30 µm; scale bars in **d-e**, 100 µm. Arrowheads indicate Tomato positive cells. Source data are provided as a Source Data file.

Supplementary Figure 4

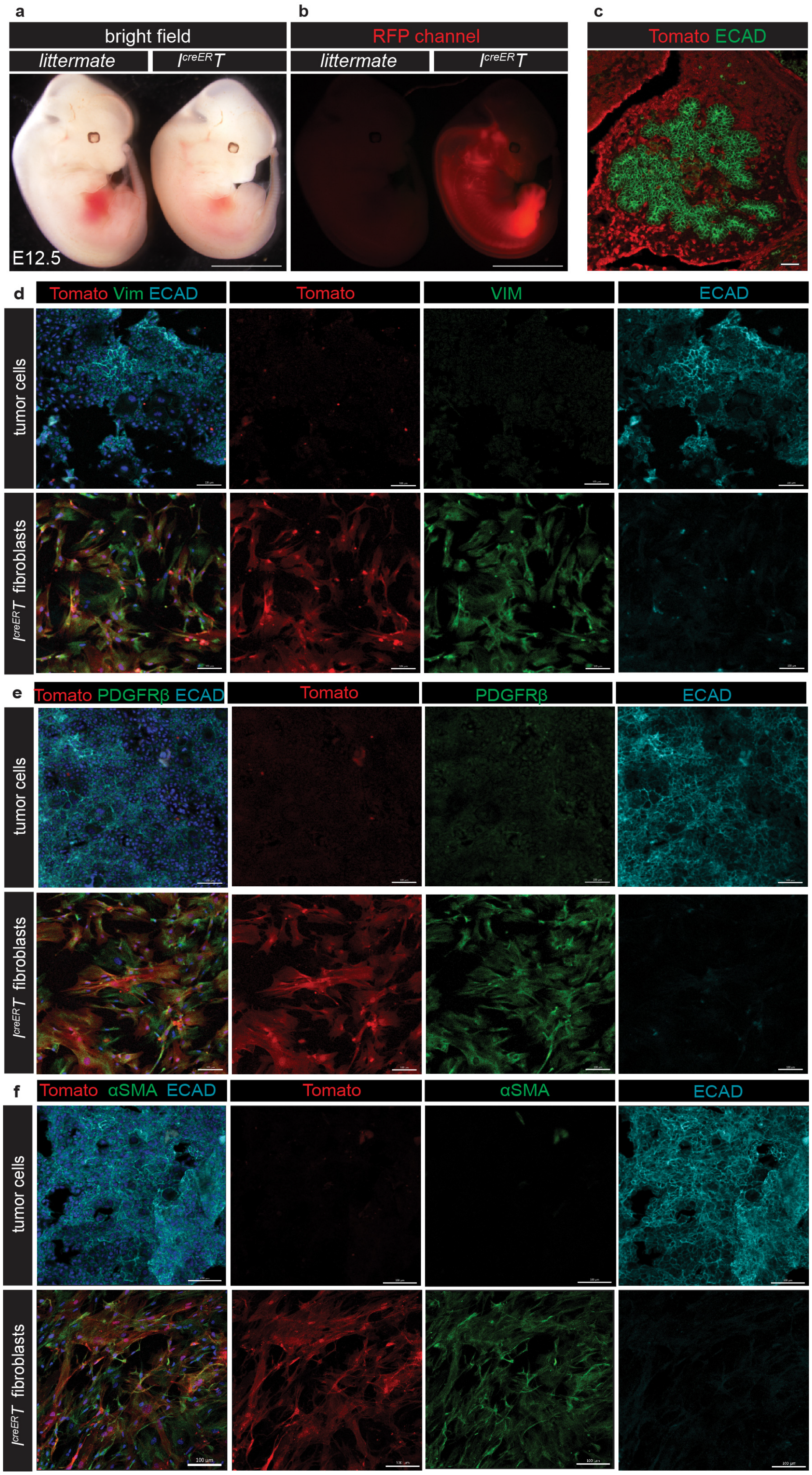

Supplementary Fig. 4. *I^creER^T* targeted fetal mesenchyme which contribute to fibroblasts, but not the epithelium in the adult pancreas. a-b Bright field and green epifluorescence views of dissected embryos of $I^{creER}T$ genotype and their littermates. Littermate n = 3 embryos; $I^{creER}T$ n = 4 embryos. Scale bar: 1000μm**. c,** Co-immunofluorescence staining on an E12.5 fetal pancreas section. n = 3 embryos. Scale bar: 100μm. **d-f**, Immunostaining on cultured fibroblasts from $I^{creER}T$ adult pancreas and tumor cells derived from the *KPF* mice. Scale bar: 100μm.

Supplementary Figure 5

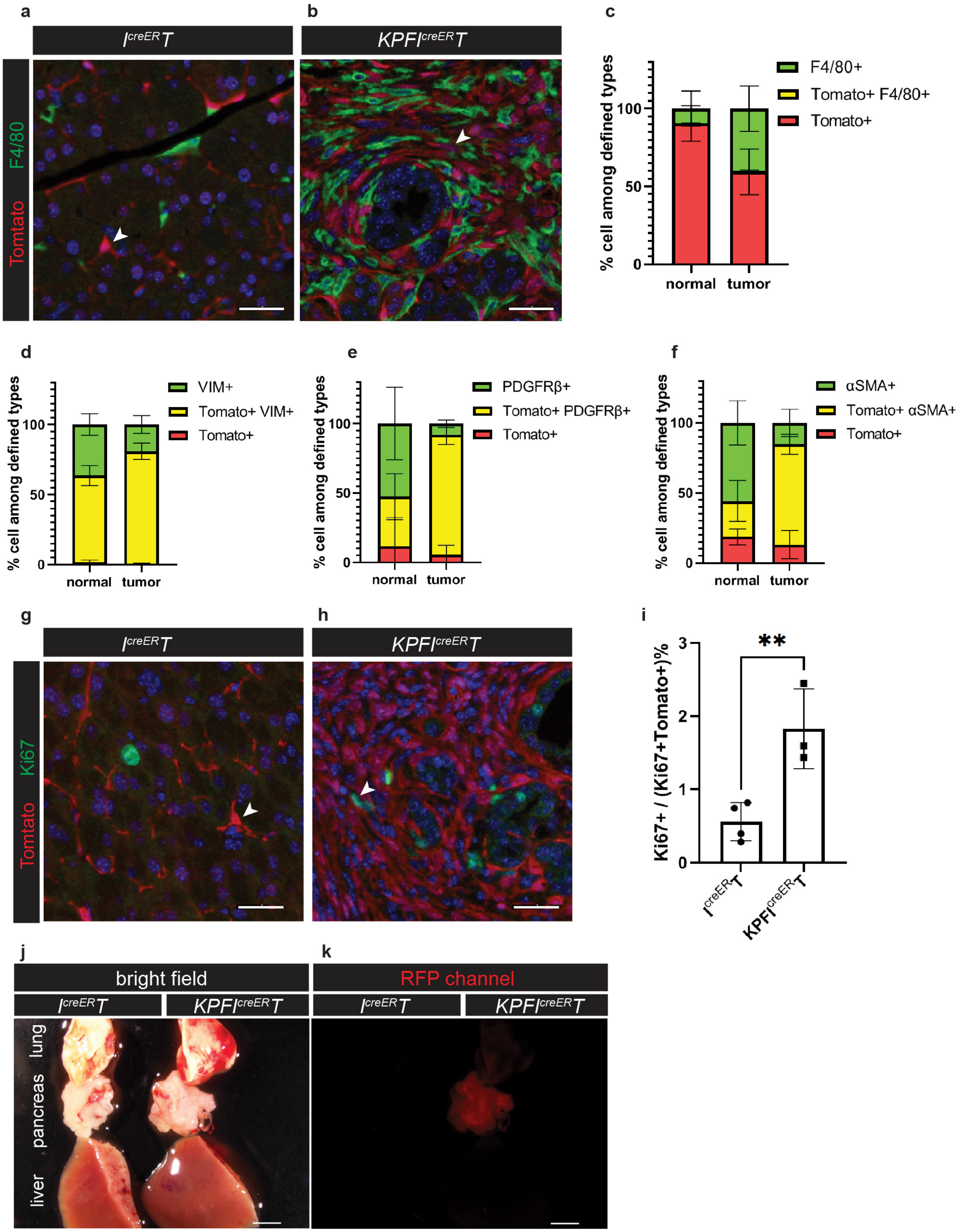

Supplementary Fig. 5. Additional characterization of the *I^{creER}T* and the *KPFI^{creER}T* models. a-c, Immunostaining on adult pancreatic tissues and quantification of cells that are either single positive or double positive for defined markers. n=5 mice for normal; n=5 mice for tumor. **d-f**, Quantification of cells that are either single positive, or double positive for defined markers in images represented in main Fig. 4**l-q**. For VIM staining, n = 4 mice for *I^{creER}T* (normal) and n = 4 mice for *KPFI^{creER}T* (tumor); for PDGFRβ, n = 5 mice for normal and n = 5 mice for the tumor; for αSMA, n = 5 mice for normal and n = 5 mice for the tumor. **g-h**, Immunostaining on pancreas sections of *I^{creER}T* and *KPFI^{creER}T* mice. **i**, Quantification of cells in images represented in G-H. n = 4 mice for *I^{creER}T* and n = 5 mice for *KPFI^{creER}T* group. Statistical significance is calculated using unpaired two-sided t-test. ** p=0.009. **j-k**, Bright field and green epifluorescence views of dissected organs. Data are mean ± SD. Scale bars in **j-k**, 1000 μm; scale bars in **a-b**, **g-h**: 30 μm. Arrowheads indicate Tomato positive cells. Source data are provided as a Source Data file.

Supplementary Figure 6

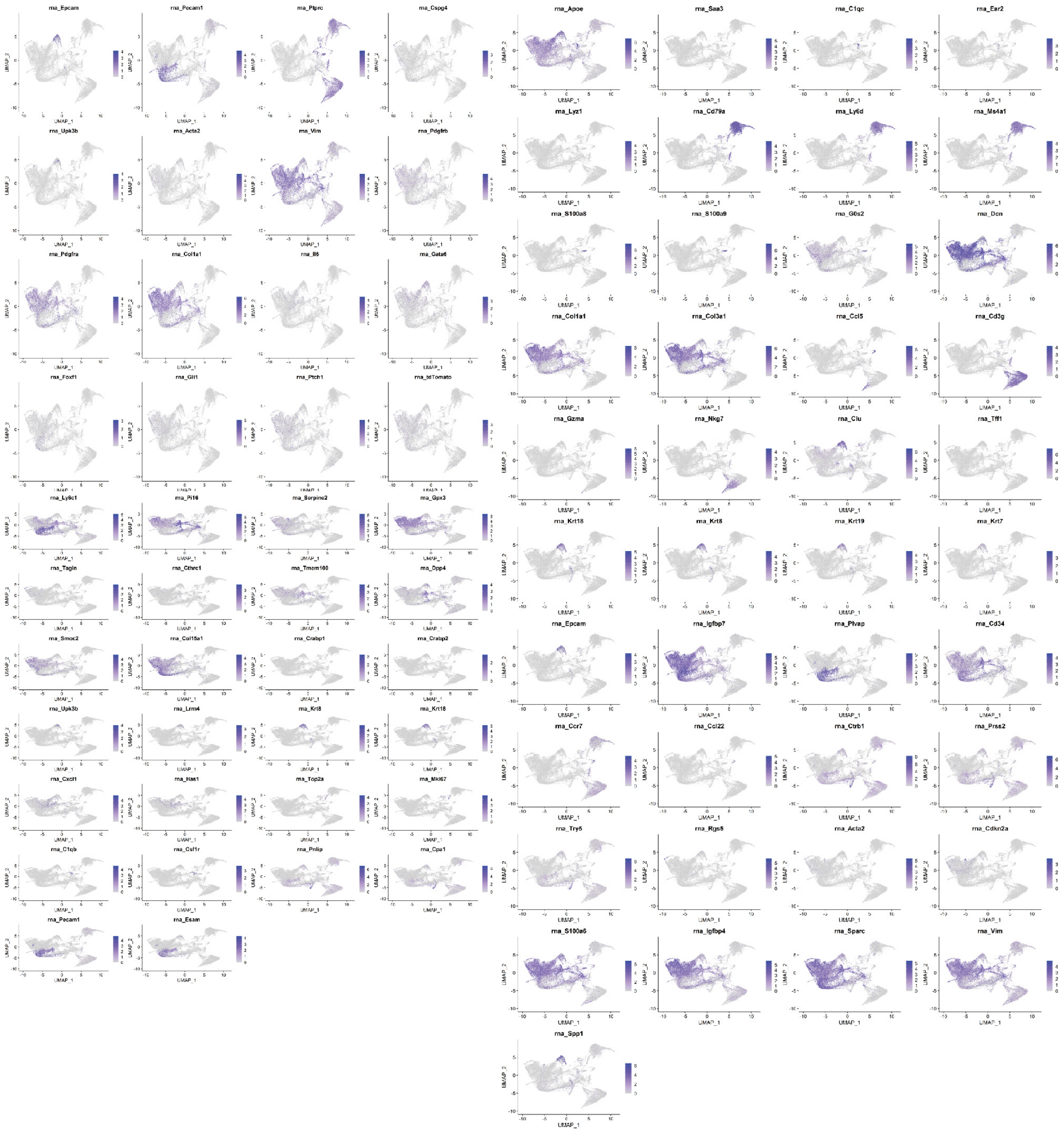

Supplementary Fig. 6. Expression of known marker genes projected on single cell clusters.

Supplementary Figure 7

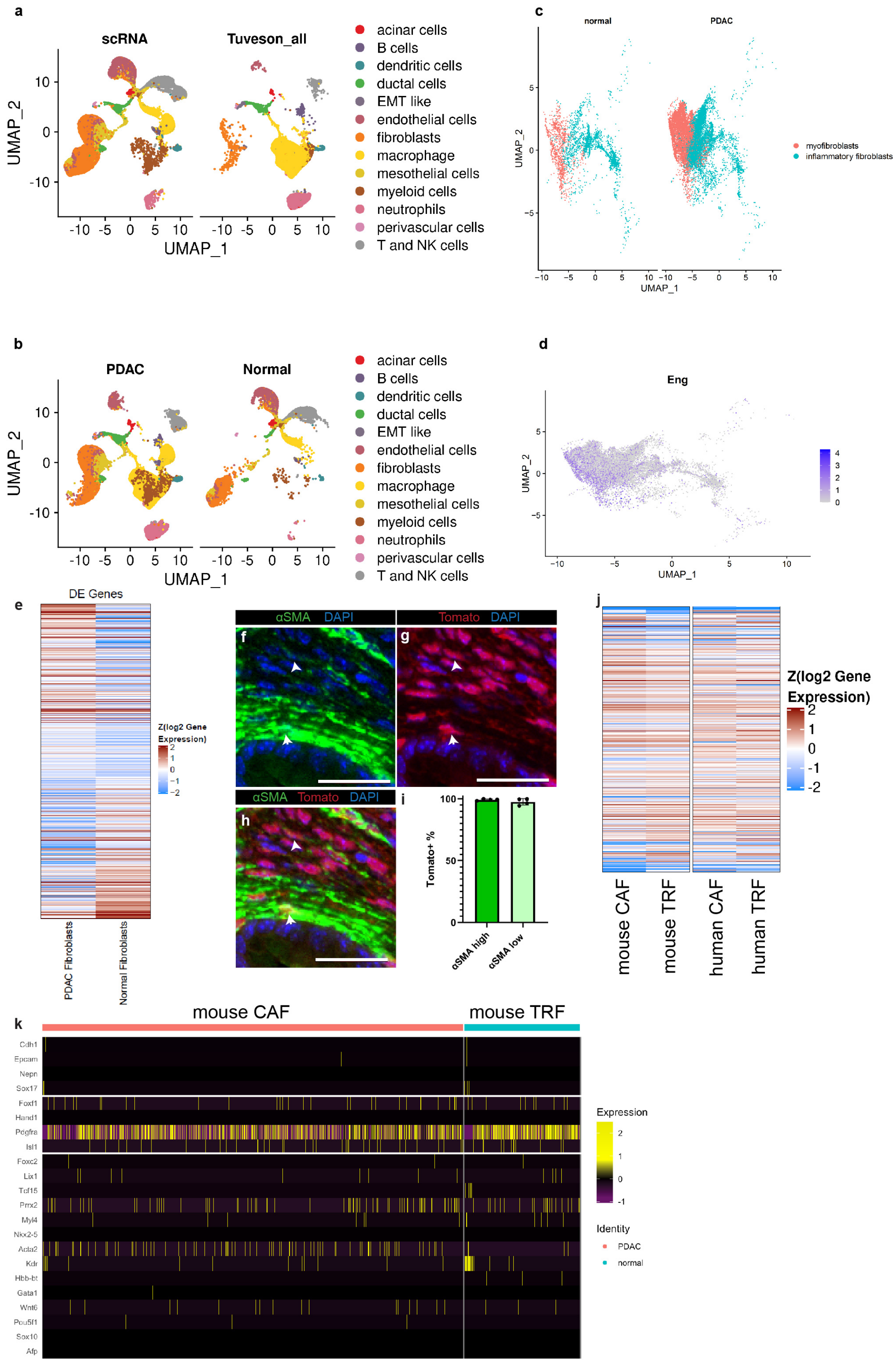

Supplementary Fig. 7. Additional bioinformatic analysis with sc-RNA seq datasets. a-b, Cells are colored according to their assigned cell type in each study, with the current study designated “scRNA” and the Tuveson mouse all cells dataset [1] designated as “Tuveson_all”. **a**, Cross comparison of the two datasets demonstrates a strong concordance. **b**, Dataset split by PDAC and normal samples, showing that there are shifts within certain cell populations between the two contexts (e.g. CAFs are more diverse than normal fibroblasts in this study). **c**, Myofibroblasts and inflammatory fibroblasts cell clusters split into normal and PDAC samples. **d**, Feature plot showing *Eng* (gene encoding for CD105) expression in fibroblasts of normal and PDAC pancreata. **e**, A heatmap of differentially expressed genes comparing normal fibroblasts and PDAC fibroblasts. **f-h**, Immunostaining of Tomato and αSMA on *KPFIT* PDAC tissue. Arrows denote an αSMA high cell (myofibroblast) and arrowheads denotes an αSMA low cell (inflammatory fibroblasts). **i**, Quantification of the percentage of Tomato+ cells within αSMA high expressing cells and αSMA low expressing cells. n=4 mice. Data are mean ± SD. **j**, Expression of fetal splanchnic gene signatures in adult mouse normal pancreatic tissue resident fibroblasts (TRFs), cancer associated fibroblasts (CAFs), human pancreatic TRFs and CAFs. **k**, Heatmap of expressions of known fetal lineage markers in adult mouse normal pancreatic and PDAC fibroblasts. Columns represent individual cells within different clusters. Scale bars in f-h: 30μm. Source data are provided as a Source Data file.

Supplementary Figure 8

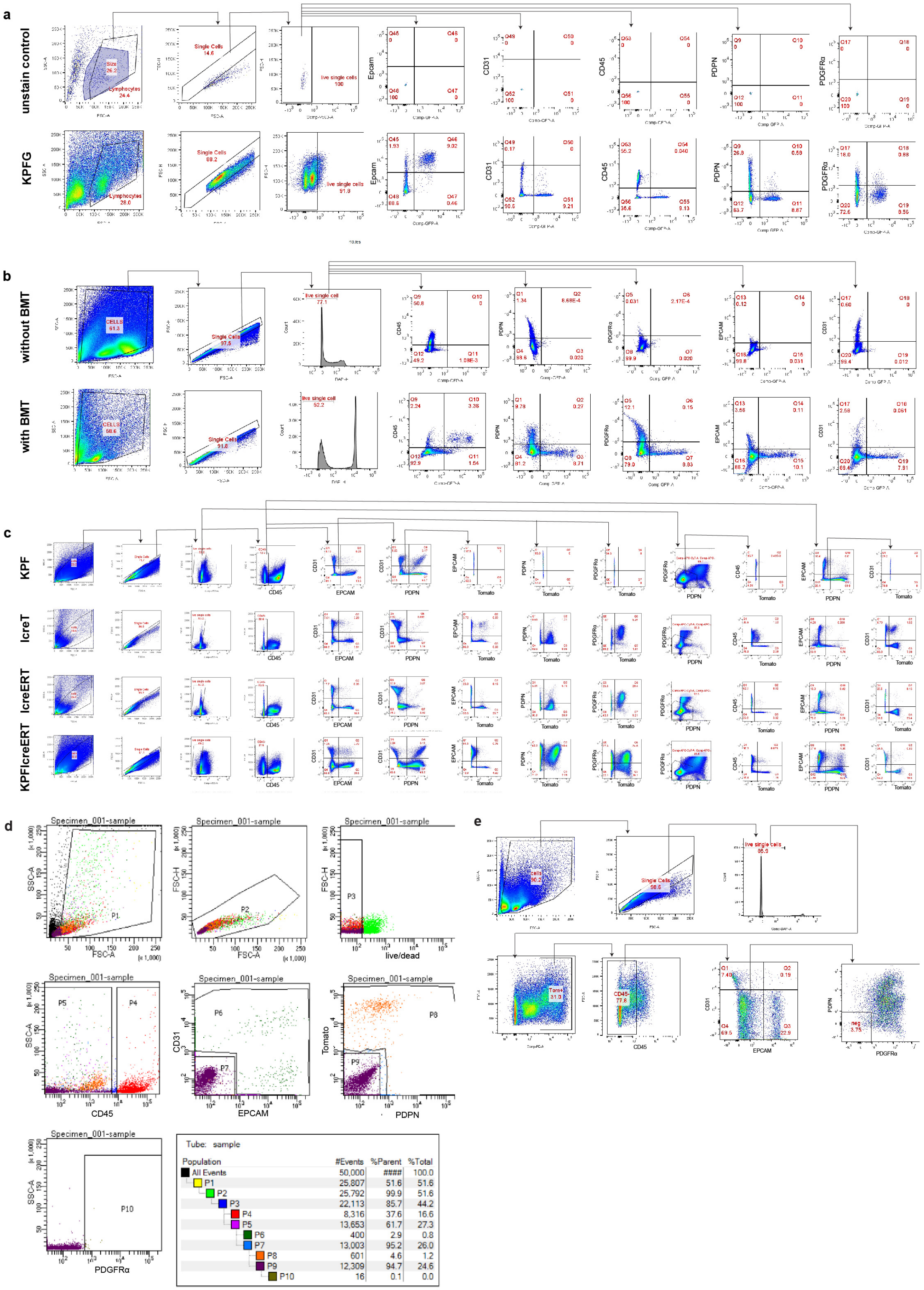

Population	#Events	%Parent	%Total
All Events	50,000	####	100.0
P1	25,807	51.6	51.6
P2	25,792	99.9	51.6
P3	22,113	85.7	44.2
P4	8,316	37.6	16.6
P5	13,653	61.7	27.3
P6	400	2.9	0.8
P7	13,003	95.2	26.0
P8	601	4.6	1.2
P9	12,309	94.7	24.6
P10	16	0.1	0.0

Supplementary Fig. 8. Gating strategies for flow cytometry analysis. **a**, Sequential gating strategy for Fig. 1e-h. **b**, Sequential gating strategy for Fig. 1m-p. **c**, Sequential gating strategy for Fig. 2l-n, Fig. 3j-l, and Fig. 4m. **d**, Sequential gating strategy for Fig. 5a. P4 was sorted as hematopoietic cells, P6 as epithelial + endothelial cells, and P8+P10 as fibroblasts. **e**, Sequential gating strategy for Supplementary Fig. 3f.

Reference

1 Elyada, E. *et al.* Cross-Species Single-Cell Analysis of Pancreatic Ductal Adenocarcinoma Reveals Antigen-Presenting Cancer-Associated Fibroblasts. *Cancer Discov* **9**, 1102-1123, doi:10.1158/2159-8290.CD-19-0094 (2019).
