# Supplementary material for: The splanchnic mesenchyme is the tissue of origin for pancreatic fibroblasts during homeostasis and tumorigenesis
Source: Nat Commun. 2023 Jan 3;14:1. doi: 10.1038/s41467-022-34464-6 (PMC9810714; doi:10.1038/s41467-022-34464-6)
Supplement: Supplementary file 3 — Description of Additional Supplementary Files [file 41467_2022_34464_MOESM3_ESM.pdf]

Description of Additional Supplementary Files

Supplementary Data 1. Differentially expressed genes comparing Tomato low (cluster 6) to Tomato low (cluster 0, 1, 13) cell clusters. Two-side Wilcoxon Rank-Sum test with Bonferroni correction was applied.

Supplementary Data 2. Differentially expressed genes comparing normal pancreatic fibroblasts to PDAC fibroblasts. Two-sided MAST test was used for statistically testing.

Supplementary Data 3. Differentially expressed genes among mouse splanchnic mesenchyme, mouse normal pancreatic fibroblasts and mouse PDAC fibroblasts. Tab “Eup.Ndw.Pup” include genes highly expressed in both the splanchnic mesenchyme and PDAC fibroblasts, compared to normal fibroblasts. “GO_Anlaysis_Eup.Ndw.Pup_NIH” tab include gene ontology analysis using genes in the “Eup.Ndw.Pup” tab. “Eup.Nup.Pdw” tab include genes highly expressed in both the splanchnic mesenchyme and normal fibroblasts, compared to PDAC fibroblasts. “GO_Anlaysis_ Eup.Nup.Pdw _NIH” tab include gene ontology analysis using genes in the “Eup.Nup.Pdw” tab. Two-side Wilcoxon Rank-Sum test with Bonferroni correction was applied.

Supplementary Data 4. Differentially expressed genes among mouse splanchnic mesenchyme, human normal pancreatic fibroblasts and human PDAC fibroblasts. Tab “Eup.Ndw.Pup” include genes highly expressed in both the splanchnic mesenchyme and PDAC fibroblasts, compared to normal fibroblasts. “GO_Anlaysis_Eup.Ndw.Pup_NIH” tab include gene ontology analysis using genes in the “Eup.Ndw.Pup” tab. “Eup.Nup.Pdw” tab include genes highly expressed in both the splanchnic mesenchyme and normal fibroblasts, compared to PDAC fibroblasts. “GO_Anlaysis_ Eup.Nup.Pdw _NIH” tab include gene ontology analysis using genes in the “Eup.Nup.Pdw” tab. Two-side Wilcoxon Rank-Sum test with Bonferroni correction was applied.

Supplementary Data 5. Antibodies used in either immunostaining on tissue sections or flow cytometry.
