# Supplementary material for: The splanchnic mesenchyme is the tissue of origin for pancreatic fibroblasts during homeostasis and tumorigenesis
Source: Nat Commun. 2023 Jan 3;14:1. doi: 10.1038/s41467-022-34464-6 (PMC9810714; doi:10.1038/s41467-022-34464-6)
Supplement: Supplementary file 9 — Reporting Summary [file 41467_2022_34464_MOESM9_ESM.pdf]

nature portfolio

Corresponding author(s): Gustavo Leone

Last updated by author(s): Oct 20, 2022

Reporting Summary

Nature Portfolio wishes to improve the reproducibility of the work that we publish. This form provides structure for consistency and transparency in reporting. For further information on Nature Portfolio policies, see our Editorial Policies and the Editorial Policy Checklist.

Statistics

For all statistical analyses, confirm that the following items are present in the figure legend, table legend, main text, or Methods section.

n/a	Confirmed	
☐	☒	The exact sample size (*n*) for each experimental group/condition, given as a discrete number and unit of measurement
☐	☒	A statement on whether measurements were taken from distinct samples or whether the same sample was measured repeatedly
☐	☒	The statistical test(s) used AND whether they are one- or two-sided<br>*Only common tests should be described solely by name; describe more complex techniques in the Methods section.*
☒	☐	A description of all covariates tested
☒	☐	A description of any assumptions or corrections, such as tests of normality and adjustment for multiple comparisons
☐	☒	A full description of the statistical parameters including central tendency (e.g. means) or other basic estimates (e.g. regression coefficient) AND variation (e.g. standard deviation) or associated estimates of uncertainty (e.g. confidence intervals)
☐	☒	For null hypothesis testing, the test statistic (e.g. *F*, *t*, *r*) with confidence intervals, effect sizes, degrees of freedom and *P* value noted<br>*Give P values as exact values whenever suitable.*
☒	☐	For Bayesian analysis, information on the choice of priors and Markov chain Monte Carlo settings
☒	☐	For hierarchical and complex designs, identification of the appropriate level for tests and full reporting of outcomes
☒	☐	Estimates of effect sizes (e.g. Cohen's *d*, Pearson's *r*), indicating how they were calculated

Our web collection on statistics for biologists contains articles on many of the points above.

Software and code

Policy information about availability of computer code

Data collection	FACS Diva v8.0.1 for flow cytometry; Vectra Polaris v1.0.13 for immunohistochemistry stainings; Zeiss Zen Black software 2.3 sp1 for imaging immunofluorescence stainings; Nikon software NIS-Elements D 5.21.02 for fresh tissue epifluorescence signal.
Data analysis	Flo Jo v9 was used for Flow cytometry data analysis. Phenochart v1.1.0 and InForm v2.6 were used for image processing and analysis following Vectra Polaris image acquisition. Imaris 9.9 was used for image analysis following Zeiss image acquisition. Raw scRNA data were processed to gene counts using CellRanger v3.1.0, followed by processing leveraging Seurat v4.0.5 through R v.4.0.2. Pathway and ontologies analysis were performed using NIH Gene Ontology resource (geneontology.org). Code used in this study is deposited in GitHub: https://github.com/KunFang93/SplMeso_PDAC_NC. The code version used to generate data in this manuscript was deposited in zenodo (https://zenodo.org/record/7150239 with DOI: 10.5281/zenodo.7150239).

For manuscripts utilizing custom algorithms or software that are central to the research but not yet described in published literature, software must be made available to editors and reviewers. We strongly encourage code deposition in a community repository (e.g. GitHub). See the Nature Portfolio guidelines for submitting code & software for further information.

Data

Policy information about availability of data

All manuscripts must include a data availability statement. This statement should provide the following information, where applicable:

- Accession codes, unique identifiers, or web links for publicly available datasets
- A description of any restrictions on data availability
- For clinical datasets or third party data, please ensure that the statement adheres to our policy

Raw and processed mouse sc-RNAseq data generated in this study have been deposited in the NCBI Gene Expression Omnibus (GEO) under accession code

GSE200903 [https://www.ncbi.nlm.nih.gov/geo/query/acc.cgi?acc=GSE200903]. The publicly available human normal pancreatic sc-RNA seq data used in this study are available in the Gene Expression Omnibus database under accession code GSE201333. [https://www.ncbi.nlm.nih.gov/geo/query/acc.cgi?acc=GSE20333] [40]. The publicly available mouse fetal foregut sc-RNA seq data used in this study are available in the Gene Expression Omnibus database under accession code GSE136689. [https://www.ncbi.nlm.nih.gov/geo/query/acc.cgi?acc=GSE136689] [33]. The publicly available mouse PDAC sc-RNA seq data used in this study are available in the Gene Expression Omnibus database under accession code GSE129455. [https://www.ncbi.nlm.nih.gov/geo/query/acc.cgi?acc=GSE129455] [10]. The raw data files are available from a public AWS S3 bucket (https://registry.opendata.aws/tabula-sapiens/)". The publicly available human PDAC sc-RNA seq data used in this study are available in the EGA database under accession EGAD00001005365. The integrated scRNA-seq objects used for analysis are provided in an online resource that can be accessed at https://fibroXplorer.com 41. Source data are provided with this paper. The remaining data are available within the Article, Supplementary Information or Source Data file.

Field-specific reporting

Please select the one below that is the best fit for your research. If you are not sure, read the appropriate sections before making your selection.

☒ Life sciences ☐ Behavioural & social sciences ☐ Ecological, evolutionary & environmental sciences

For a reference copy of the document with all sections, see nature.com/documents/nr-reporting-summary-flat.pdf

Life sciences study design

All studies must disclose on these points even when the disclosure is negative.

Sample size	Power and sample size calculations were performed using PASS 2008 version 08.0.13.
Data exclusions	No data that passed appropriate quality controls was excluded in the analysis.
Replication	Each experiment was replicated at least three times and all attempts at replication were successful. Flow cytometry analysis was performed using multiple flow instruments at both the Medical University of South Carolina and the College of Wisconsin.
Randomization	Immuno-staining images were randomized during processing and quantification. For bone marrow transplantation experiments, mice were randomly allocated into different groups. For other experiments, mice were allocated into different groups based on defined genotypes.
Blinding	Investigators were blinded to the groups during image quantification. For other experiments, blinding was not possible as mice of defined genotypes need to be assigned to different groups.

Reporting for specific materials, systems and methods

We require information from authors about some types of materials, experimental systems and methods used in many studies. Here, indicate whether each material, system or method listed is relevant to your study. If you are not sure if a list item applies to your research, read the appropriate section before selecting a response.

Materials & experimental systems

n/a	Involved in the study	
☐	☒	Antibodies
☐	☒	Eukaryotic cell lines
☒	☐	Palaeontology and archaeology
☐	☒	Animals and other organisms
☒	☐	Human research participants
☒	☐	Clinical data
☒	☐	Dual use research of concern

Methods

n/a	Involved in the study	
☒	☐	ChIP-seq
☐	☒	Flow cytometry
☒	☐	MRI-based neuroimaging

Antibodies

Antibodies used	Tomato: Rockland, 600-401-379; GFP: CST, 2555S; aSMA: CST, 14968S; Pdgfrb: CST, 3169S; Vimentin: CST, 5741S; E-cadherin: CST, 3195S; Keratin 19: Hybridoma Bank Iowa, TOMA-III; Isl1: Hybridoma Bank Iowa, 39.4D5; Pdx1: Abcam, ab47267; CD45: CST, 70257S; F4/80: CST, 70076S; Ki67: Abcam, ab16667; Pdpn, Biolegend, 127417; Pdgfra: ThermoFisher, 17-1401-81; CD45: ThermoFisher, 25-0451-81; CD31: Fisher Scientific, 565509; Epcam: Biolegend, 118225; CD31: Biolegend, 102522; Foxf1: R&D system, AF4798. Secondaries: Omnimap anti rabbit, Roche, 760-4311; Omnimap anti goat, Roche, 760-4311; Omnimap anti rat, Roche, 760-4457; Omnimap anti mouse, Roche, 760-4310; donkey anti rabbit, 488, ThermoFisher, A21206; donkey anti rat 594, invitrogen, A21209; donkey anti mouse, 488, Thermo Fisher, A32766; donkey anti rabbit, 594, Thermo Fisher, A32754; donkey anti rat, 488, ThermoFisher, A21208; donkey anti goat, 647, Thermo Fisher, A32849
Validation	These antibodies have been validated by the manufacturers. Whenever possible, negative control samples or cells were used for further validation.<br>Validation Statements from Companies:

Tomato: Rockland:
Assay by immunoelectrophoresis resulted in a single precipitin arc against anti-Rabbit Serum and purified and partially purified Red Fluorescent Protein (Discosoma). No reaction was observed against Human, Mouse or Rat serum proteins.
GFP: CST,
GFP Antibody detects GFP, YFP, and CFP-tagged proteins exogenously expressed in cells. This antibody does not detect RFP-tagged proteins. Please note that the GFP, YFP, and CFP tags add approximately 27 kDa to the molecular weight of the fusion protein. All Species Expected
aSMA: CST, 14968S
Western blot analysis of extracts from various tissue using α-Smooth Muscle Actin Antibody and GAPDH (D16H11) XP® Rabbit mAb #5174. As expected, α-skeletal muscle extracts are negative for smooth muscle actin.
Pdgfrb: CST, 3169S
Western blot analysis of extracts from various cell lines, using PDGF Receptor β (28E1) Rabbit mAb. his antibody reacts to endogenous mouse protein.
Vimentin: CST, 5741S
Western blot analysis of extracts from various cell lines using Vimentin (D21H3) XP® Rabbit mAb. This antibody reacts to endogenous mouse protein.
E-cadherin: CST, 3195S
Immunohistochemical analysis of paraffin-embedded mouse prostate using E-Cadherin (24E10) Rabbit mAb.
Keratin 19: Hybridoma Bank Iowa, TOMA-III
Immunohistochemical analysis of paraffin-embedded mouse biliary tract.
Isl1: Hybridoma Bank Iowa, 39.4D5
Validated for immunofluorescence staining and immunohistochemistry. Tested positive for human and mouse.
Pdx1: Abcam, ab47267
PDX1 antibody LS-C427194-GOS12 is an HRP-conjugated rabbit polyclonal antibody to PDX1 (aa36-85) from mouse. It is reactive with human, mouse, rat and other species. Validated for IHC and WB.
CD45: CST, 70257S
Immunohistochemical analysis of paraffin-embedded 4T1 mammary tumor using CD45 (D3F8Q) Rabbit mAb performed on the Leica® BOND™ Rx. Reacts with mouse endogenous protein.
F4/80: CST, 70076S
Immunohistochemical analysis of paraffin-embedded mouse liver using F4/80 (D2S9R) XP® Rabbit mAb.
Ki67: Abcam, ab16667
Suitable for: Flow Cyt (Intra), IHC-P, WB, mIHC, ICC. Knockout validated. Reacts with: Mouse, Rat, Human
Pdpn, Biolegend, 127417
Mouse thymic epithelial stromal cell line TE-71 stained with Podoplanin (clone 8.1.1) APC/Cyanine7 or unstained.
Pdgfra: ThermoFisher, 17-1401-81
Flow staining of NIH-3T3 cells with 0.5 µg of Rat IgG2a K Isotype Control APC (Product # 17-4321-81) or 0.5 µg of Anti-Mouse CD140a (PDGF Receptor a) APC.
CD45: ThermoFisher, 25-0451-81
Staining of BALB/c splenocytes with 0.06 µg of Rat IgG2b K Isotype Control PE-Cyanine7 (Product # 25-4031-82) or 0.06 µg of Anti-Mouse CD45 PE-Cyanine7.
CD31: Fisher Scientific, 565509
Flow cytometric analysis of CD31 expression on mouse bone marrow cells.
Epcam: Biolegend, 118225
Flow cytometry quality tested.
CD31: Biolegend, 102522
Flow cytometry quality tested. Verified reactivity to mouse.
Foxf1: R&D system, AF4798
Detects mouse and human Foxf1 in Western blots.

Eukaryotic cell lines

Policy information about cell lines

Cell line source(s)	Primary cells lines were generated from mouse pancreata at the Medical University of South Carolina.
Authentication	Cell lines were examined by marker expressions for authentication.
Mycoplasma contamination	The cell lines have not been tested for Mycoplasma contamination.
Commonly misidentified lines (See ICLAC register)	NA

Animals and other organisms

Policy information about studies involving animals; ARRIVE guidelines recommended for reporting animal research

Laboratory animals	Species: C57BL6, FVB, and Black Swiss. Sex: both males and females. Age: embryonic day 7.5-birth. Day 21-200. Strains: Pdx1Flpo/+; KrasG12D/+;p53frt/+, R26GFP, UBC-GFP, R26Tomato, Isl1cre, Isl1creER
Wild animals	The study did not involve wild animals.

Field-collected samples	The study did not involve field-collected samples.
Ethics oversight	Institutional Animal Care and Use Committee at the Medical University of South Carolina. Medical College of Wisconsin Institutional Animal Care and Use Committee

Note that full information on the approval of the study protocol must also be provided in the manuscript.

Flow Cytometry

Plots

Confirm that:

- [x] The axis labels state the marker and fluorochrome used (e.g. CD4-FITC).
- [x] The axis scales are clearly visible. Include numbers along axes only for bottom left plot of group (a 'group' is an analysis of identical markers).
- [x] All plots are contour plots with outliers or pseudocolor plots.
- [x] A numerical value for number of cells or percentage (with statistics) is provided.

Methodology

Sample preparation	Normal pancreas: Dissociation media for normal pancreas is Collagenase P (1 mg/ml), Soybean Trypsin inhibitor (0.2 mg/ml), Dnase I (12 U/ml), HEPES (10 mM), Rock inhibitor Y (10 µM) in HBSS (with Ca2+). Pancreas was dissected, minced in Eppendorf tubes, and then transferred to a Miltenyi gentleMACS tube with 2.5ml of dissociation media. Dissociation was performed on a gentleMACS Dissociator using program 37m-TDK-1 for 15 min. 15ml of cold DMEM+10% fetal bovine serum (FBS) dissociation mix was then filtered through a 70 µm and then a 100 µm membrane. Red blood cell lysis buffer was then added to the pellate and pipetted. After centrifuge, cells are suspended in FACS buffer (5% FBS, 10 µM Y, 20 mM HEPES, 5 mM MgCl2, 12 U/ml DNase I in PBS). After Fc block, cells were incubated with fluorophore conjugated antibodies.<br><br>Tumor pancreas: Dissociation media for tumor bearing pancreas was prepared using the Miltenyi gentleMACS tumor dissociation kit. Pancreas was dissected and cut into small pieces using a razor blade. A full program of 37m-TDK-1 was used to dissociate the tumor. Procedures after dissociation were the same as the normal pancreas.
Instrument	BD Fortessa X-20 and FACSAria III
Software	FACS Diva for data acquisition and Flow Jo for data analysis.
Cell population abundance	Around 70-80% purity, determined by secondary sorting of the sorted samples.
Gating strategy	FSC/SSC gates were drawn to eliminate dead cells and small debris. Negative/Positive gates were drawn based on negative control samples and distinct population shapes.

- [x] Tick this box to confirm that a figure exemplifying the gating strategy is provided in the Supplementary Information.
